# Photooxidative molecular damage under blue light

**DOI:** 10.1038/s12276-025-01609-8

**Published:** 2026-01-07

**Authors:** Eojin Kim, Seoyoon Kim, Minseung Kim, Duyoung Min

**Affiliations:** 1 Department of Chemistry, Ulsan National Institute of Science and Technology, Ulsan, Republic of Korea; 2 X-Dynamic Research Center, Ulsan National Institute of Science and Technology, Ulsan, Republic of Korea

**Keywords:** Mitochondria, Homeostasis, DNA damage and repair, Cell death, Ageing

## Abstract

The widespread adoption of artificial lighting has substantially increased human exposure to blue light across various environments, raising concerns about its potential adverse effects on human health. Over the past decades, blue light-induced biological responses have been investigated across multiple levels—from mechanistic studies of photoinduced reactive oxygen species generation to broader physiological consequences. Since all cellular and tissue-level effects ultimately originate from structural and functional alterations in molecular components, a comprehensive understanding of blue light-induced molecular damage is clearly warranted. This review summarizes current knowledge and recent findings on photooxidative molecular damage induced by blue light exposure, with a focus on the primary photochemical mechanisms of reactive oxygen species generation, blue light-sensitive endogenous photosensitizers, and the resulting oxidative damage to key biomolecules, including proteins, DNA and lipids. These insights collectively establish a more integrated framework for understanding how blue light compromises molecular integrity within cells.

## Blue light exposure linked to molecular damage in humans

Sunlight is essential for life on Earth, comprising ultraviolet (UV), visible and infrared radiation. While the harmful effects of UV on the skin and eyes are well established, recent attention has turned to blue light—the high-energy portion of visible light—which is abundant in sunlight and also commonly emitted by digital screens and LED lighting. Experimental studies in human cells and animal models have shown that excessive blue light exposure can generate reactive oxygen species, leading to oxidative damage of DNA, proteins and lipids. Such molecular damage may contribute to skin photoaging and has been implicated as a potential factor in retinal disorders, including age-related macular degeneration.

This summary was initially drafted using artificial intelligence, then revised and fact-checked by the author.

## Introduction: blue light as a potential hazard to human health

Life on Earth is regularly exposed to sunlight, which serves as the primary natural source of light. The solar spectrum reaching Earth’s surface primarily comprises ultraviolet (UV; ~280–400 nm), visible (~400–750 nm) and infrared (>750 nm) radiation^1,2^. Photons, the quantum particles of light, carry energy (*E*) that is inversely proportional to their wavelength (*λ*), as described by Planck’s equation (*E* = *hc*/*λ*), where *h* is Planck’s constant and *c* is the speed of light; that is, shorter wavelengths correspond to higher photon energy. In light-exposed human tissues, photons are absorbed by endogenous photosensitizers (PSs), initiating a cascade of photochemical and biochemical reactions that can lead to either beneficial effects (for example, vitamin D synthesis) or harmful effects (for example, photooxidative damage) on human health^3–5^.

UV radiation, with the highest energy in the solar spectrum, has long been considered the most hazardous component to human tissues, particularly the skin, owing to its well-documented role in DNA damage, photoaging and increased skin cancer risk^6^. Therefore, extensive research and public health efforts have focused on UV protection, leading to the widespread use of sunscreen, protective clothing and UV-filtering eyewear. However, in modern times, lifestyle and technological advancements have considerably altered human light exposure patterns. On average, most individuals spend ~90% of their lifetime indoors^1^, where artificial light sources, such as light-emitting diode (LED) lighting and digital screens, have largely replaced natural sunlight as the primary source of illumination^7^. Unlike sunlight, which provides a full spectrum of wavelengths from UV to infrared radiation, artificial light sources emit narrower spectral bands, predominantly within the visible range, though some also produce UV or infrared radiation depending on the light source type^7^. As a result, scientific and public health research has increasingly focused on the potential health effects of visible light exposure.

Among the various wavelengths in the visible spectrum, blue light has garnered notable attention for its impact on human health for several reasons, as outlined below.

**Fig. 1:**
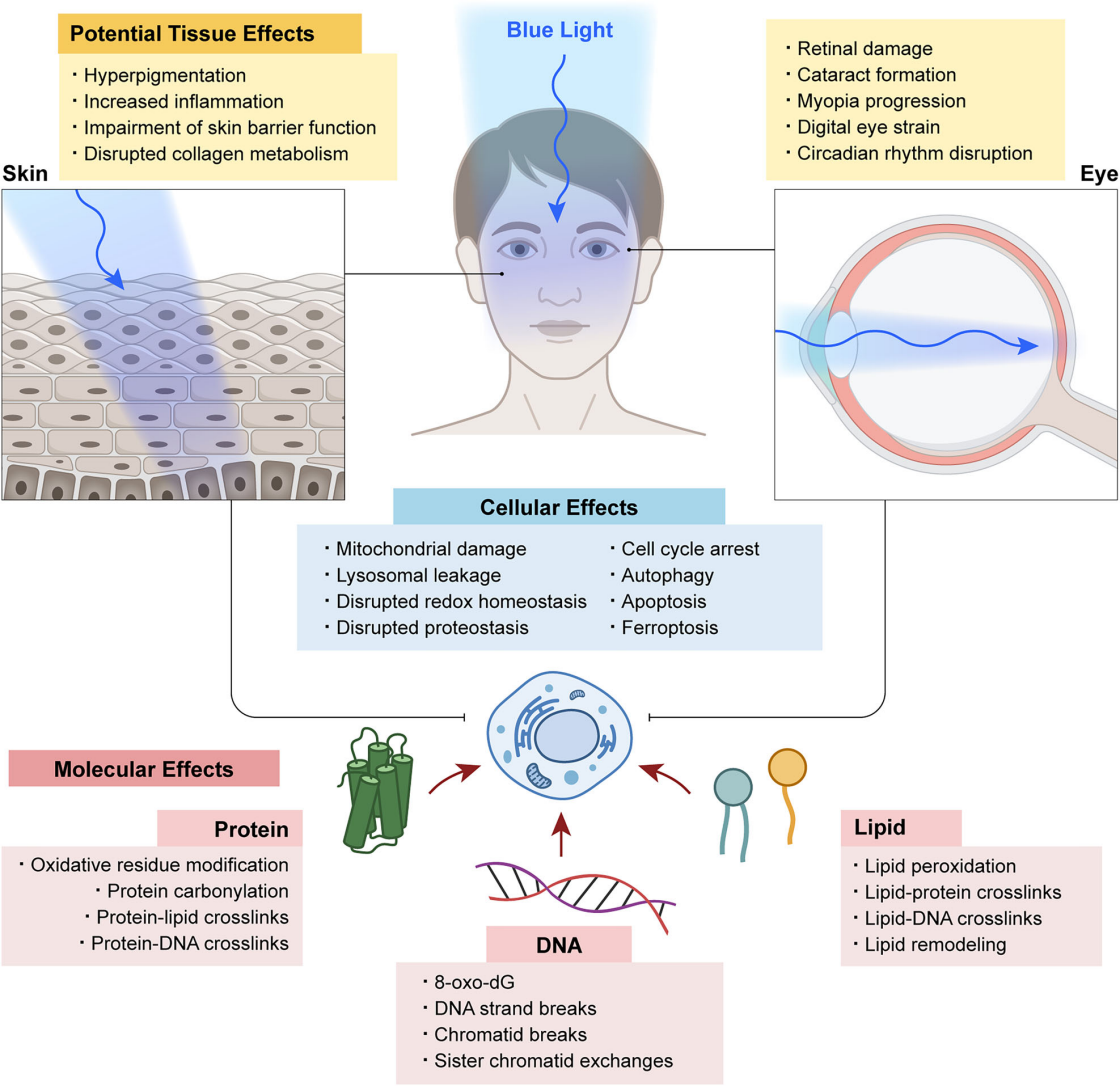
Multilevel biological effects of blue light exposure on human skin and eyes. Schematic overview of the molecular, cellular and tissue-level effects of blue light exposure on human skin and eyes. Molecular effects include reported damage to key biomolecules such as proteins, DNA and lipids.

1. Blue light, categorized into blue–violet (~400–450 nm) and blue–turquoise (~450–500 nm)^1^, has a shorter wavelength than other visible wavelengths and, therefore, the highest photon energy in the visible spectrum. In addition, unlike UV radiation, which is largely absorbed by the outer layers of the skin and eyes, blue light penetrates deeper into these organs^1,4,8^. In the eye, for example, it reaches the retina, where it contributes to visual processing via classical photoreceptor cells (rods and cones) and modulates nonvisual pathways through intrinsically photosensitive retinal ganglion cells involved in circadian rhythm regulation^9^.
2. Blue LEDs, with a peak emission in the range of ~440–460 nm, are widely used in LED-based white lighting (phosphor-converted white LEDs) for general illumination and in display backlighting (RGB white LEDs) for digital devices such as smartphones, tablets, TVs and computer monitors^7^. The widespread adoption of LED-based lighting and displays in recent years has led to consistent and prolonged exposure to blue light, raising concerns about its effects on eye and skin health, sleep regulation and overall well-being.
3. Although clinical studies involving human participants remain relatively limited, extensive research has shown that blue light exposure adversely affects ocular and skin tissues, based primarily on studies using relevant human cells, tissues and animal models^1,8,10^ (Fig. 1 and Table 1). For instance, potential ocular effects include retinal damage, an increased risk of age-related macular degeneration, cataract formation, myopia progression, and digital eye strain—characterized by fatigue, dryness and irritation—as well as circadian rhythm disruption^1,9–12^. In terms of skin health, potential effects of blue light exposure include hyperpigmentation, increased inflammation, impairment of the skin barrier function, and disrupted collagen metabolism, which may contribute to wrinkle formation^8^.

**Table 1:** Molecular damage under blue light or visible light containing blue wavelengths.

Molecule	Damage type	Experimental model	Light exposure condition	Ref
Protein	Oxidative modification of amino acids and peptide residues	Amino acids, polypeptides	150 W incandescent light, up to 30 h	^156^
	Oxidative modification of protein residues	Lysozyme with riboflavin supplemented	449 nm, 9×10^5 ^J/mol; 400–700 nm, 7.5 mW/cm^2^, up to 3 h	^158^
		Lysozyme and α-lactalbumin with riboflavin supplemented	452 nm, 2.0 W/m^2^, up to 4 h	^159^
		HeLa, B16F10 and iHCEC cells with flavin derivatives supplemented	450 nm, 14 mW/cm^2^, 10 min	^46^
		Drosophila eye tissues	465 nm, 2 mW/cm^2^, 8 h	^152^
	Oxidative modification of protein residues (mainly through O_2_-confined photooxidation)	Maltose-binding protein, carbonic anhydrase	447 nm, 9.2 or 16 mW/cm^2^, up to 12 h	^19^
	Oxidative modification of protein residues (protein carbonylation)	Human RPE cells with lipofuscin supplemented	390–550 nm, 2.8 mW/cm^2^, up to 48 h	^164^
	Protein adducts with dicarbonyl metabolites	ARPE-19 cells with A2E supplemented, albino rats	(ARPE-19 cell) 430 nm, 1.5 mW/cm^2^, 20 min; (Rat) 430 nm, 1 mW/cm^2^, 2 h/day for 7 days	^165^
DNA	DNA strand breaks	Isolated calf thymus DNA, HeLa cells	415–465 nm, 1.7 mW/cm^2^, 2 h	^169^
		Human fibroblasts with bilirubin supplemented	420–490 nm, 16.7 W/m^2^, up to 50 kJ/m^2^, up to 50 min	^79^
		KB cells	400–700 nm, 141 μW/cm^2^, 15.2 kJ/m^2^; 450 nm, 652 μW/cm^2^, up to 70.4 kJ/m^2^	^170^
		Zebrafish, ARPE-19 cells	440 nm, 3.7 ± 0.75 mW/cm^2^, 24 h	^140^
	8-Oxo-2′-deoxyguanosine (8-oxo-dG)	Isolated calf thymus DNA with riboflavin supplemented	100 W tungsten lamp, up to 4 min	^173^
		Human lung fibroblasts with riboflavin supplemented, mouse lymphoma cells	150 W tungsten lamp, up to 30 min	^174^
		AS52 cells	400–500 nm, 1–4 W/m^2^, up to 150 kJ/m^2^; 400, 420, 455 and 475 nm, 4 W/m^2^/nm, 20 J/m^2^	^175^
		FEK4, TK6 and GLL19 cells	405 nm, 600 kJ/m^2^, 90–120 min; 434 nm, 1,000 kJ/m^2^, 90–120 min	^176^
	DNA strand breaks, 8-oxo-dG	P3, HaCaT, L1210 and AS52 cells	400–500 nm, up to 42 kJ/m^2^	^171^
		HaCaT cells	466 nm, 204 W/m^2^, up to 82 min	^86^
	DNA strand breaks, 8-oxo-dG, cyclobutene pyrimidine dimers, micronucleus formation	Human keratinocytes	415 nm, 8 mW/cm^2^, up to 14.4 J/cm^2^	^178^
	DNA crosslinks, DNA–protein crosslinks (DPCs)	Mouse lung and leukemia cells, human fibroblast and embryonic cells	400–700 nm, 4.6 or 8.6 W/m^2^, up to 20 h	^183^
		P3 cells	405 nm, 400 W/m^2^, up to 2,628 kJ/m^2^; 434 nm, 600 W/m^2^, up to 4,018 kJ/m^2^	^184^
			405 nm, up to 1,525 kJ/m^2^	^185^
	DNA crosslinks, DPCs, chromatid breaks, chromatid exchanges	Mouse embryonic and lung cells	400–700 nm, 4.6 W/m^2^, up to 20 h	^182^
	Chromatid breaks	Mouse embryos	400–700 nm, 4.6 W/m^2^, up to 20 h	^186^
	Sister chromatid exchanges	V-79 cells	420–500 nm, 3.61 W/m^2^, up to 300 kJ/m^2^	^187^
		Human lymphocytes	480 nm, 4,350 lux, up to 6 h	^188^
	Sister chromatid exchanges, endoreduplicated chromosome	CHO and UV135 cells	425–475 nm, 1–5 μW/cm^2^/nm, up to 24 h	^138^
	DNA strand breaks, chromatin condensation	B16F1 cells	465 nm, up to 50 W/m^2^, up to 24 h	^142^
	Enhanced DNA lesions in mitochondria	Human RPE cells	390–550 nm, 2.8 mW/cm^2^, up to 6 h	^50^
		SCC-25 cells	420 nm, up to 70 mW/cm^2^, up to 84 J/cm^2^	^136^
	Enhanced DNA lesions in mitochondria, 8-oxo-dG	HaCaT cells	408, 466, and 478 nm, 4.77–20.40 mW/cm^2^, up to 50–190 J/cm^2^; 400–700 nm, 40 mW/cm^2^, 30–100 J/cm^2^	^177^
Lipid	Lipid peroxidation, lipid-derived reactive carbonyl species (RCS)	Isolated lipofuscin, melanolipofuscin and melanosomes	408–495 nm, 220 mW/cm^2^, up to 70 min	^88^
		Linoleic acid and DHA with lipofuscin, melanolipofuscin or melanosomes supplemented	Argon ion laser (488 and 514 nm irradiation mixed at ~1:1 ratio), 50–1,500 mW/cm^2^, 10 min; 400–700 nm, 112 mW/cm^2^, up to 20 min	^206^
		Linoleic acid, squalene, oleic acid, human skin tissues	430 nm, 1–10 mW/cm^2^, 10 min	^146^
		Human RPE cells with lipofuscin supplemented	390–550 nm, 2.8 mW/cm^2^, up to 48 h	^110^
		ARPE-19 cells with A2E supplemented, C57BL/6 mice	430 nm, 50 mW/cm^2^, 10 klux (ARPE-19 cell) and 3.25 klux (mouse), 30 min	^108^
		ARPE-19 cells	400–510 nm, 18 mW/cm^2^ (for 410–500 nm), up to 1.5 h	^147^
		ARPE-19 cells, albino rats	435–445 nm, 11.2 W/m^2^, up to 12 h	^151^
		ARPE-19 cells, C57BL/6 mice	Blue light LED with peaks at 434.4 and 421.2 nm, 1,500 lux, 24 h (ARPE-19 cell) and 12:12 blue light:dark cycle for 14 days (mouse)	^149^
			(ARPE-19 cell) 440 ± 15 nm, 400–700 nm, 200 ± 20 lux, up to 36 h; (mouse) 440 ± 15 nm, 800 ± 20 lux, 12:12 light:dark cycle, up to 7 days	^150^
		HaCaT cells, hairless mice (C57BL/6 × albino), human volunteers	460 nm, 44 mW/cm^2^, up to 10 min;400–480 nm, 11 mW/cm^2^, 10 min	^48^
		C57BL/6 mice	410 ± 10 nm, 29.2 mW/cm^2^, up to 100 J/cm^2^, twice daily up to 10 days	^144^
				^199^
		Bovine subcutaneous preadipocytes	460–475 nm, 2 mW/cm^2^, 12 h	^137^
		Frog retina and rod outer segment	455 and 494 nm, up to 1.6 klux, 30 min	^207^
		Wistar rats	460 nm, 400 mW/cm^2^, 15 min	^148^
		Albino rats	White light LED with peaks at 449, 484, 551 and 664 nm, 12:12 light: dark cycle, up to 7 days	^139^
	Lipid-derived RCS, RCS–protein adducts	Isolated lipofuscin, ARPE-19 cells with lipofuscin supplemented	400–500 nm, 2.8 mW/cm^2^, 48 h	^167^
		ARPE-19 cells with A2E supplemented, bovine retina extract	430 nm, 3 mW/cm^2^; 400–700 nm, 5 or 7 mW/cm^2^	^145^
		Bovine RPE cells	470 nm, 4.8 mW/cm^2^, 1–50 J/cm^2^	^166^
		Albino rats	400–700 nm, 1 or 5 klux, 3 h	^197^
			400–700 nm, 5 klux, 3 h	^204^
	Lipid composition change	COS-7 fibroblasts	465 nm, 1 mW/cm^2^, up to 1 h	^210^
		Albino rats	400–700 nm, 1,075–1,345 lux, up to 72 h	^208^
			490–580 nm, 1,750–2,000 lux, up to 72 h	^209^
			490–580 nm, up to 24 h	^211^

Blue light exposure can induce diverse and complex histological and metabolic effects^5,8,10,13–15^, which vary depending on the specific tissue type and its manifestations, as briefly outlined above. The most widely recognized chemical mechanism underlying these effects is photooxidative damage to biomolecules, such as DNA, proteins and lipids, primarily driven by light-induced reactive oxygen species (ROS)^5,8,10,13–16^. Furthermore, unlike photomechanical and photothermal damage^15^—which typically occur under high-intensity light exposure, such as during laser therapy—photooxidative damage can also result from prolonged exposure to low or moderate light intensity levels^1,7,8,10^. Prolonged blue light exposure may therefore lead to a redox imbalance, favoring oxidants over antioxidants, which in turn promotes the accumulation of molecular damage and disrupts relevant metabolic pathways^17^. Consequently, photooxidative molecular damage via ROS is considered the most relevant chemical mechanism underlying the broad phototoxic outcomes associated with blue light exposure.

## Photochemical pathways of light-induced ROS generation

Blue light-induced oxidative damage begins with the absorption of photons by endogenous blue light-sensitive PSs in human tissues, such as the skin and eyes. These PSs include flavins, porphyrins and other chromophores capable of absorbing blue light (discussed in detail in the following section). Upon photon absorption, electrons within these PSs are excited to a higher energy state. The excited PSs then facilitate the generation of ROS from dissolved molecular oxygen (O_2_). ROS are highly reactive derivatives of O_2_, including superoxide radical (O_2_^•–^), hydrogen peroxide (H_2_O_2_), hydroxyl radical (^•^OH) and singlet oxygen (^1^O_2_)^3–5^. ROS can diffuse across cellular compartments and tissues, propagating oxidative damage even beyond the initial site of photochemical reactions^18^.

**Fig. 2:**
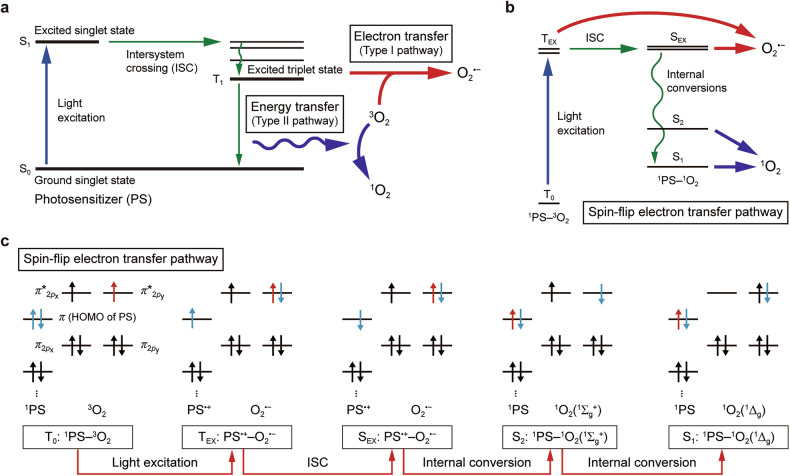
Photochemical pathways of light-induced ROS generation. **a** An energy diagram illustrating the conventional type I and type II pathways for ROS generation. A PS is photoexcited from the ground singlet state (S_0_) to an excited singlet state (S_1_), followed by ISC to an excited triplet state (T_1_). In this state, the PS interacts with molecular oxygen (O_2_, also denoted as ^3^O_2_ in its triplet ground state), producing ROS such as superoxide radical (O_2_^•–^) or singlet oxygen (^1^O_2_) via electron transfer (type I) or energy transfer (type II), respectively. **b**, **c** An energy diagram (**b**) and molecular orbital diagram (**c**) of the spin-flip electron transfer pathway for ROS generation. This pathway occurs through transient, short-range interactions between PS and O_2_ (~3.5 Å), forming the ground triplet state (T_0_) of the PS–O_2_ complex. Upon blue light excitation, the complex transitions to an excited triplet state (T_EX_), where electron transfer from the PS to O_2_ generates O_2_^•–^ as an intermediate ROS. The T_EX_ state subsequently undergoes ISC to an excited singlet state (S_EX_) via electron spin-flip in the PS. The S_EX_ state then relaxes through internal conversions to lower excited singlet states (S_2_ and S_1_), where the corresponding oxygen species are singlet oxygen (O_2_(^1^Σ_g_^+^) and O_2_(^1^Δ_g_)), respectively. The latter is commonly referred to as ^1^O_2_. ROS generated during this process may dissociate from the PS at each stage. This pathway is illustrated based on a recent study in which the PS is Trp and the excitation wavelength lies within the blue light range (400–500 nm) (ref. ^19^). Panels **b** and **c** are adapted with permission from ref. ^19^.

ROS-generating photochemical processes can be primarily classified into three major pathways^3,19–21^: the conventional type I and type II pathways, and the recently identified spin-flip electron transfer pathway (Fig. 2). In both the type I and II pathways (Fig. 2a), photon absorption excites electrons in PSs from the ground singlet state (S_0_) to an excited singlet state (S_1_), which has a relatively short lifetime in the nanosecond range^22,23^. The singlet state refers to an electronic state in which all electron spins are paired, resulting in a total spin quantum number S = 0. The S_1_ state then undergoes intersystem crossing (ISC) to a more stable, longer-lived excited triplet state (T_1_) via spin-orbit coupling. The T_1_ state is characterized by two electrons occupying different molecular orbitals with parallel (unpaired) spins, leading to a total spin quantum number S = 1. PSs in the T_1_ state typically have a microsecond-to-millisecond lifetime, providing sufficient time for molecular interactions^22–24^. The longer lifetime of T_1_-state PSs, combined with the spin conservation rule, enables efficient ROS production through interactions with O_2_ in its ground triplet state (^3^O_2_, also denoted as O_2_(^3^Σ_g_^–^)).

In the type I pathway, excited PSs engage in electron transfer reactions with O_2_, generating radical species such as O_2_^•–^ (ref. ^3^). The conversion of O_2_^•–^ into other ROS can rapidly occur through catalytic reactions under physiological conditions. For example, O_2_^•–^ undergoes enzyme-catalyzed dismutation to generate H_2_O_2_. H_2_O_2_, a relatively mild ROS, can further decompose into ^•^OH via the Fenton reaction or the Haber–Weiss reaction, both of which are iron-catalyzed processes^25^. ^•^OH is highly reactive, exhibiting rapid reaction kinetics, and can induce severe oxidative damage to nearby biomolecules in a relatively nonspecific manner^26–28^. In the type II pathway, excited PSs transfer their energy directly to O_2_, leading to the formation of ^1^O_2_, also denoted as O_2_(^1^Δ_g_) (ref. ^22^). This ROS, ^1^O_2_, corresponds to the lowest excited state of O_2_. ^1^O_2_ is a highly reactive electrophilic species that preferentially reacts with electron-rich moieties, such as double bonds in the hydrocarbon tails of unsaturated lipids and aromatic rings of amino acid residues in proteins^2,29–31^.

The spin-flip electron transfer pathway represents a unique ROS-generating mechanism, involving multiple electron transfer reactions between a PS and O_2_, as well as electron spin-state inversion during these reactions^19,20^ (Fig. 2b,c). This mechanism occurs only during transient, short-distance interactions between PS and O_2_ within ~3.5 Å, forming the ground triplet state (T_0_) of the PS–O_2_ molecular complex. Upon photon absorption, the PS–O_2_ complex transitions to an excited triplet state (T_EX_), where electron transfer from the PS to O_2_ generates O_2_^•–^ as an intermediate ROS. The T_EX_ state then undergoes ISC to an excited singlet state (S_EX_) via electron spin-flip in the PS. Subsequently, the S_EX_ state relaxes through internal conversion to a lower excited singlet state (S_2_), during which an electron is transferred back from O_2_ to the PS, yielding singlet oxygen (O_2_(^1^Σ_g_^+^)). Further internal conversion leads to the lowest excited singlet state (S_1_), forming the more stable form of singlet oxygen (O_2_(^1^Δ_g_)), which is commonly referred to as ^1^O_2_. Thus, the spin-flip electron transfer pathway results in the production of both radical and nonradical ROS, O_2_^•–^ and ^1^O_2_, respectively.

## Blue light-sensitive endogenous PS

**Fig. 3:**
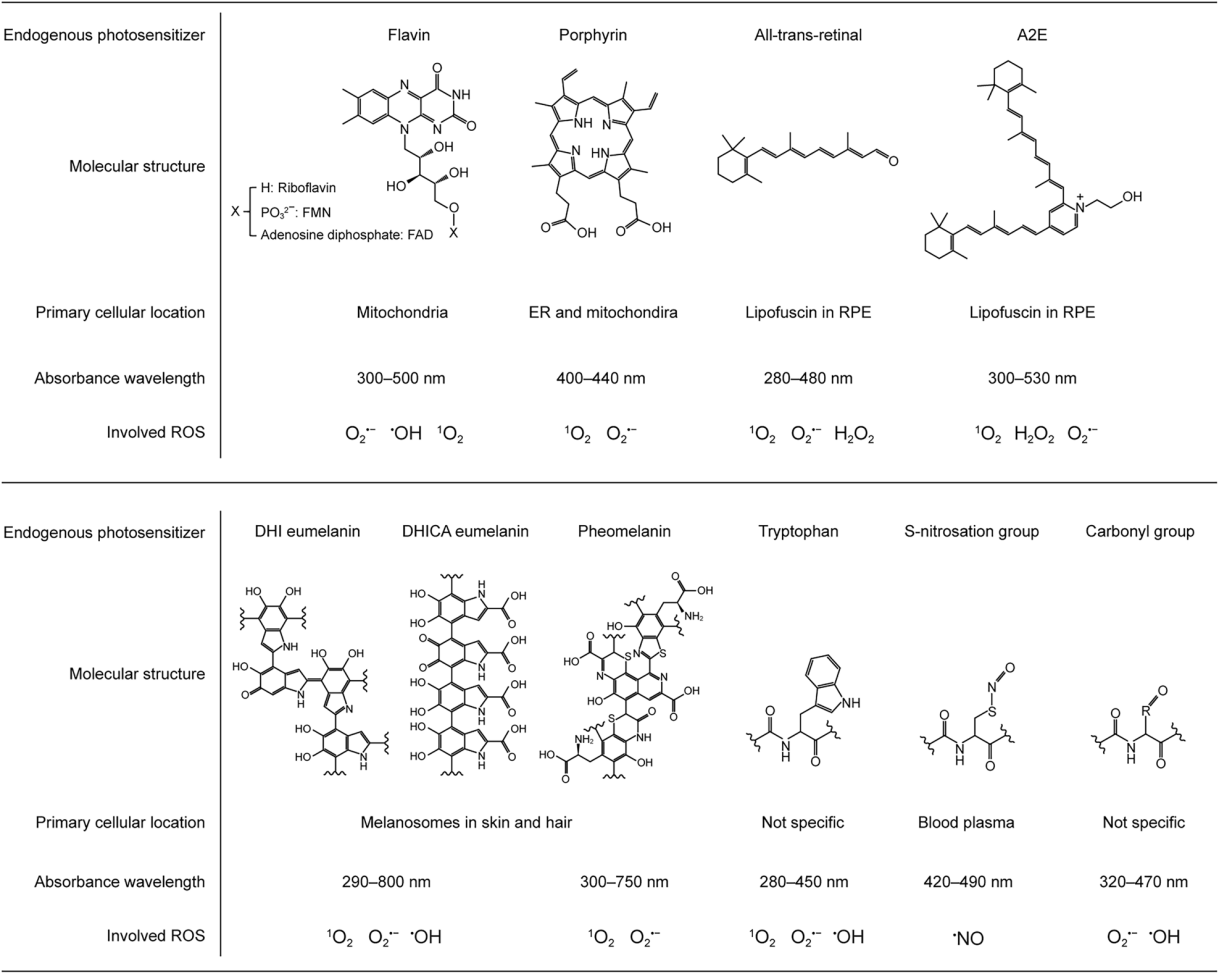
Endogenous PSs capable of blue light absorption. Endogenous PSs discussed in the main text are summarized in terms of their molecular structures, primary cellular locations, absorbance wavelength ranges and the types of ROS they can generate under blue light exposure. The absorbance range of eumelanin is derived from assembled DHI/DHICA eumelanin complexes^115^. The absorbance ranges for tryptophan, *S*-nitrosation group and carbonyl group refer to those of free tryptophan^19^, *S*-nitosoalbumin^129^ and carbonylated bovine serum albumin^135^, respectively. A2E, N-retinylidene-N-retinylethanolamine; DHI, dihydroxyindole; DHICA, 5,6-dihydroxyindole-2-carboxylic acid; ER, endoplasmic reticulum; RPE, retinal pigment epithelium; O_2_^•–^, superoxide radical; H_2_O_2_, hydrogen peroxide; ^•^OH, hydroxyl radical; ^1^O_2_, singlet oxygen; ^•^NO, nitric oxide.

Endogenous PSs are naturally synthesized cellular molecules that absorb light in the UV–visible spectrum and are involved in essential physiological functions^4,32,33^. Their photoactivity properties and phototoxicity thresholds vary across tissue and cell types, reflecting differences in the cellular microenvironment and the subcellular localization of these PSs^8,10,12,34,35^. This section provides an overview of major endogenous PSs that may contribute to photooxidative stress in tissues exposed to blue light (Fig. 3).

### Flavins

Flavins, including riboflavin (vitamin B₂) and its derivatives flavin mononucleotide and flavin adenine dinucleotide, are major endogenous chromophores that absorb blue light through their isoalloxazine ring, with a peak absorption around 450 nm (refs.^36–38^). Flavins are widely distributed in cells as enzyme cofactors: the human genome encodes approximately 90 flavoproteins^39^, most of which are localized in the mitochondria and catalyze redox reactions in metabolic pathways such as the citric acid cycle and fatty acid oxidation^39,40^. In particular, flavin adenine dinucleotide and flavin mononucleotide are highly enriched in mitochondria-rich tissues, such as the retina^5,41,42^. Retinal cells, including those of the neural retina and retinal pigment epithelium (RPE), are metabolically highly active, with elevated oxygen consumption and increased ROS production as a byproduct of cellular respiration^40,43,44^.

Flavin derivatives and flavoproteins can promote the generation of ROS, such as O_2_^•–^, ^•^OH and ^1^O_2_, contributing to mitochondrial dysfunction under blue light exposure^41,43,45–51^. These ROS may arise either from direct interactions between photoexcited flavins and O_2_ (refs.^41,45^), or from enhanced electron leakage in the mitochondrial electron transport chain under blue light^41,43,48,51^. One mechanistic basis for flavin-mediated ROS generation involves light-induced changes in the multiple redox states of flavins^36,41,51–54^. Upon light excitation, flavins can undergo photoreduction through interactions with nearby amino acid residues, yielding highly reactive one-electron and two-electron reduced species^32,36,41,54^. The one-electron reduced flavin radical anion can subsequently react with O_2_ to form O_2_^•–^ or accept a proton from biomolecular radical cations or other proton donors to yield a neutral flavin radical^36,41,52,53,55^. These neutral radicals may further react with O_2_ to generate flavin peroxyl radicals and subsequently form hydroperoxides. Meanwhile, the two-electron reduced flavin species can either transfer energy to O_2_ to generate ^1^O_2_ (ref. ^41^) or react with O_2_ to form flavin peroxide species^56,57^.

### Porphyrins

Porphyrins constitute another class of endogenous chromophores that absorb blue light. These molecules, often metalated with iron to form heme, exhibit absorption bands in the blue–violet range, with the Soret band typically appearing around 400–440 nm (refs.^33,58,59^). The Soret band originates from π → π* transitions within the conjugated aromatic ring system of porphyrin^59,60^. The exact position and intensity of the Soret band can vary depending on the central metal ion, which influences the electronic structure and symmetry of the porphyrin^58,61^. Upon blue light exposure, porphyrin molecules can populate the triplet-excited state, which predominantly generates ^1^O_2_ via the type II photochemical pathway^32^. Consequently, porphyrins have been widely used in the development of type II PSs for photodynamic therapy in cancer treatment^62,63^.

The human genome encodes 192 heme-binding proteins^64^, which are primarily located in the endoplasmic reticulum (ER) and mitochondria. In the mitochondrial inner membrane, heme groups within cytochromes play a critical role in the electron transport chain. Multiple studies have indicated that the heme-containing proteins can be activated by blue light, enhancing electron transport and ROS production in retinal and epidermal tissues^42,43,65^. Cytochrome c oxidase, a component of mitochondrial complex IV, contains heme a and exhibits absorption maxima near 420 nm (oxidized) and 440 nm (reduced), making it susceptible to blue light exposure^5,66^. It has been identified as a suppressor of retinal metabolism following in vivo blue light exposure (404 nm), leading to reduced oxygen consumption, which is expected to promote ROS generation via electron leakage^67^. In addition, mitochondrial cytochrome P450 family members, such as Cyp1b1 and Cyp1a1—which contain heme b—have been shown to respond to 450 nm blue light^68^. Their activation under blue light irradiation induces the generation of ROS such as O_2_^•–^, which functions as a signaling mediator in regulating scar remodeling during the skin wound healing process^68^.

In addition to the conventional photochemical pathways mentioned above, alternative mechanisms have also been proposed. For instance, when oxidized cytochrome c (Cyt-c), containing ferric iron (Fe^3+^), is exposed to blue light, the formation of ferrous iron (Fe^2+^) has been detected at its iron-porphyrin center^69^. The blue light-induced reduction of Cyt-c enhances electron transfer from complex III to Cyt-c, leading to mitochondrial hyperpolarization and elevated levels of ROS such as O_2_^•–^ (ref. ^69^). Another characteristic of iron-bound porphyrins is their ability to serve as carriers for diatomic gaseous molecules, including O_2_, carbon monoxide (CO) and nitric oxide (NO)^59^. Especially, NO is a radical compound and a source of reactive nitrogen species (RNS)^70^. It has been reported that blue light illumination at 450 nm can trigger the release of NO from nitrosyl complexes of hemoglobin^71^ and iron–sulfur clusters in mitochondria^72^. The released NO may further contribute to oxidative or nitrosative stress pathways^70,73,74^.

Hemoproteins, particularly hemoglobin, are degraded by the reticuloendothelial system^75–77^. In this catabolic pathway, heme is oxidized by heme oxygenase-1, producing the linear tetrapyrrole biliverdin. Biliverdin is subsequently reduced to bilirubin by biliverdin reductase, which is considered the final metabolite of heme catabolism. Bilirubin absorbs blue light around 450 nm (refs.^32,78^), leading to the generation of ROS, such as ^1^O_2_, O_2_^•–^ and H_2_O_2_ (refs.^32,78–80^). Thus, it acts as an endogenous PS that is responsive to blue light. However, bilirubin is also known to be vulnerable to ROS and can undergo self-photooxidation owing to its intrinsic antioxidant properties^81–83^. Therefore, bilirubin alone may not play a dominant role in blue light-induced molecular damage.

### Lipofuscin

Lipofuscin is an age-related pigment that accumulates in RPE and keratinocytes, as well as in cardiac and neuronal tissues, under both physiological and pathological conditions^84–86^. Chemically heterogeneous, lipofuscin consists of highly oxidized proteins (30–70%), lipids (20–50%), carbohydrates (4–7%) and metal cations (~2%, particularly iron), with its composition and photochemical properties varying across different tissues^32,85^. Lipofuscin exhibits autofluorescence, characterized by absorption across the UV to visible blue–green wavelength range^85^. The photochemical activity of lipofuscin in RPE and keratinocytes supports its role as an endogenous PS, capable of generating ROS (for example, ^1^O_2_, O_2_^•–^ and H_2_O_2_)—albeit with relatively low quantum yields—upon blue light exposure^86–88^. Within the RPE, all-trans-retinal (ATR) and *N*-retinylidene-*N*-retinylethanolamine (A2E) are well-characterized chromophores of lipofuscin^32,89–92^. These compounds have been found to accumulate in the cells of patients with age-related macular degeneration and Stargardt disease, contributing to increased oxidative stress and cytotoxicity under blue light exposure^12,90,93–96^.

ATR, the aldehyde derivative of vitamin A, serves as a major chromophore at high concentrations in the retina^89,90,96,97^. In darkness, opsin in photoreceptor cells covalently bind 11-*cis*-retinal. Upon light stimulation, 11-*cis*-retinal is isomerized to ATR, which then dissociates from opsin^90,96,98^. Under normal physiological conditions, ATR is recycled back to 11-*cis*-retinal within the RPE via the visual cycle^90,96,98^. However, intense light exposure or defects in retinal metabolism can lead to the accumulation of free ATR in the photoreceptor outer segments^95,96^. ATR exhibits a broad absorption spectrum ranging from 280 nm to 480 nm, with a peak absorbance at 380 nm (refs.^89,99^). Since visible light penetrates through all ocular layers and reaches the retina^32,97^, accumulated ATR effectively acts as a blue-light PS, contributing to retinal photodamage^95–97,100^. In its triplet-excited state, ATR can transfer energy to O_2_, generating ^1^O_2_ (refs.^89,100^), which can in turn induce lipid oxidation in photoreceptor membranes^89,100^. This energy transfer mechanism has also been supported by time-dependent density functional theory calculations^89^. In addition, blue light-irradiated ATR can generate O_2_^•–^ and H_2_O_2_, probably through type I photochemical pathways^100^. Physiologically, accumulated ATR exacerbates retinal oxidative stress by upregulating NADPH oxidase, which in turn increases O_2_^•–^ levels^94^. The ATR dimer series represents another important class of lipofuscin chromophores, including the ATR dimer, ATR dimer–ethanolamine and ATR dimer–phosphatidylethanolamine, which exhibit absorbance maxima at 432 nm, 510 nm and 511 nm, respectively^92^. This dimer series also contributes to RPE phototoxicity owing to its susceptibility to photooxidation under blue light exposure^92^.

A2E is a bisretinoid compound with a conjugated polyene structure, consisting of a pyridinium headgroup connected to two retinoid arms^91,101,102^. It is formed as a byproduct of vitamin A cycling in RPE cells^101^. A2E displays two distinct absorption peaks: one in the UV region (~336 nm) and another in the blue region (~439 nm)^99,101,102^. Under blue light, photochemical reactions involving A2E generate ROS, such as ^1^O_2_, H_2_O_2_ and O_2_^•–^ (refs.^103–105^). The resulting oxidative stress can disrupt the regulation of calcium homeostasis^13^ and alter gene expression by promoting the release of apoptotic factors^13,106,107^. In addition, photoactivated A2E perturbs iron homeostasis, inducing Fenton-type reactions and triggering ferroptosis^108^. However, the relative contribution of A2E to lipofuscin-mediated photosensitized damage remains controversial^96,109^. Comparative studies of its photophysical and photochemical properties show that A2E irradiated at 407 nm consumes ~20 times less oxygen than ATR, primarily for ^1^O_2_ generation^104^. Although A2E produces O_2_^•–^ approximately four times faster than ATR, its quantum yield is lower than that for ^1^O_2_ generation and is about 30-fold lower than that of riboflavin^104^. Furthermore, physiological concentration of A2E in RPE cells is probably insufficient to account for blue light-induced phototoxicity^99,110^.

### Melanins

Melanins are classified into five types based on the chemical properties of their polymeric pigments^32,111^: eumelanin, pheomelanin, neuromelanin, allomelanin and pyomelanin. Among them, eumelanin (brown–black) and pheomelanin (red–yellow) are the most abundant endogenous chromophores in the skin and hair^85,112^. Both pigments are derived from tyrosine through oxidative polymerization, resulting in complex conjugated structures^32,111^. Tyrosine itself is a known endogenous PS—along with tryptophan and phenylalanine—capable of generating ROS upon UV exposure owing to its aromatic structure^113,114^. Eumelanin consists of dihydroxyindole (DHI) and 5,6-dihydroxyindole-2-carboxylic acid (DHICA) subunits, which are biosynthesized from tyrosine^32,111^. It exhibits a broad absorption spectrum, strongest in the UV range (290–320 nm) but extending into the near-infrared region (~800 nm)^32,115^. Pheomelanin, on the other hand, contains benzothiazine units from cysteine incorporation and absorb photons in both the UV and visible ranges (300–750 nm)^115^.

Although melanin is widely recognized for its photoprotective functions^116,117^, such as quenching ROS and shielding biomolecules from UV-induced damage, recent studies have uncovered its ROS-generating photochemical reactivity (refs.^112,118–123^). Melanin localized within melanosomes, the physiological site of its function, has been examined for such properties. Under blue (400–500 nm)^118,119,122,123^ and green (532 nm)^120,121^ light exposure, melanin acts as a PS, producing O_2_^•–^ and ^1^O_2_, which subsequently contribute to its own oxidative degradation^124^. The resulting degradation products further amplify blue light-induced O_2_^•–^ generation^93,125^. In addition, eumelanin in melanosomes promotes Fenton-type reactions, thereby facilitating ^•^OH production and initiating melanin degradation and lipid peroxidation, even in the absence of blue light^122,126^. Given the heterogeneous nature of melanin, synthetic analogs have been used to assess the photoactivity of individual moieties. For instance, DHICA–melanin exhibits greater ^1^O_2_ generation upon blue light exposure (402–508 nm), whereas DOPA–melanin is more effective at producing O_2_^•–^ (ref. ^127^). Synthetic pheomelanin, particularly variants rich in carboxylated benzothiazine units, generates higher levels of O_2_^•–^ than noncarboxylated forms upon UV–blue light exposure (390–490 nm)^128^, and also produces ^1^O_2_ more efficiently than synthetic eumelanin under green light (532 nm)^120,124^.

### Modified functional groups and amino acid residues in proteins

Certain protein modifications can introduce endogenous chromophores that absorb blue light and induce photochemical reactions involving the generation of RNS or ROS. Two notable examples are S-nitrosated proteins and carbonylated proteins. S-nitrosated proteins contain an S-nitrosation bond, formed between a cysteine thiol (–SH) and an NO moiety^33^. These *S*-nitroso compounds, such as *S*-nitroso-albumin found in human plasma, can function as blue light-sensitive chromophores^33^. They typically absorb in the 420–490 nm region, where blue light induces cleavage of the S–NO bond^33,129^, releasing NO and contributing to the formation of other RNS^70,129^. Carbonylated proteins, a well-known hallmark of oxidative protein damage^130–132^, result from the addition of carbonyl (C=O) groups via direct photosensitized oxidation and reactive carbonyl species^130,132–134^. These carbonyl moieties can function as chromophores that absorb UVA and blue light (320–470 nm), generating O_2_^•–^ and ^•^OH, probably via the type I photochemical pathway^135^. Tryptophan (Trp), an aromatic amino acid, has recently been identified as an intrinsic blue light-absorbing PS at ~450 nm, capable of generating multiple types of ROS, including ^1^O_2_ and ^•^OH (ref. ^19^). Notably, Trp residues buried within proteins can contribute to the generation of these ROS, primarily damaging the protein interior through a unique mechanism known as O_2_-confined photooxidation^19^ (see the following section for details).

## Photooxidative molecular damage under blue light exposure

**Fig. 4:**
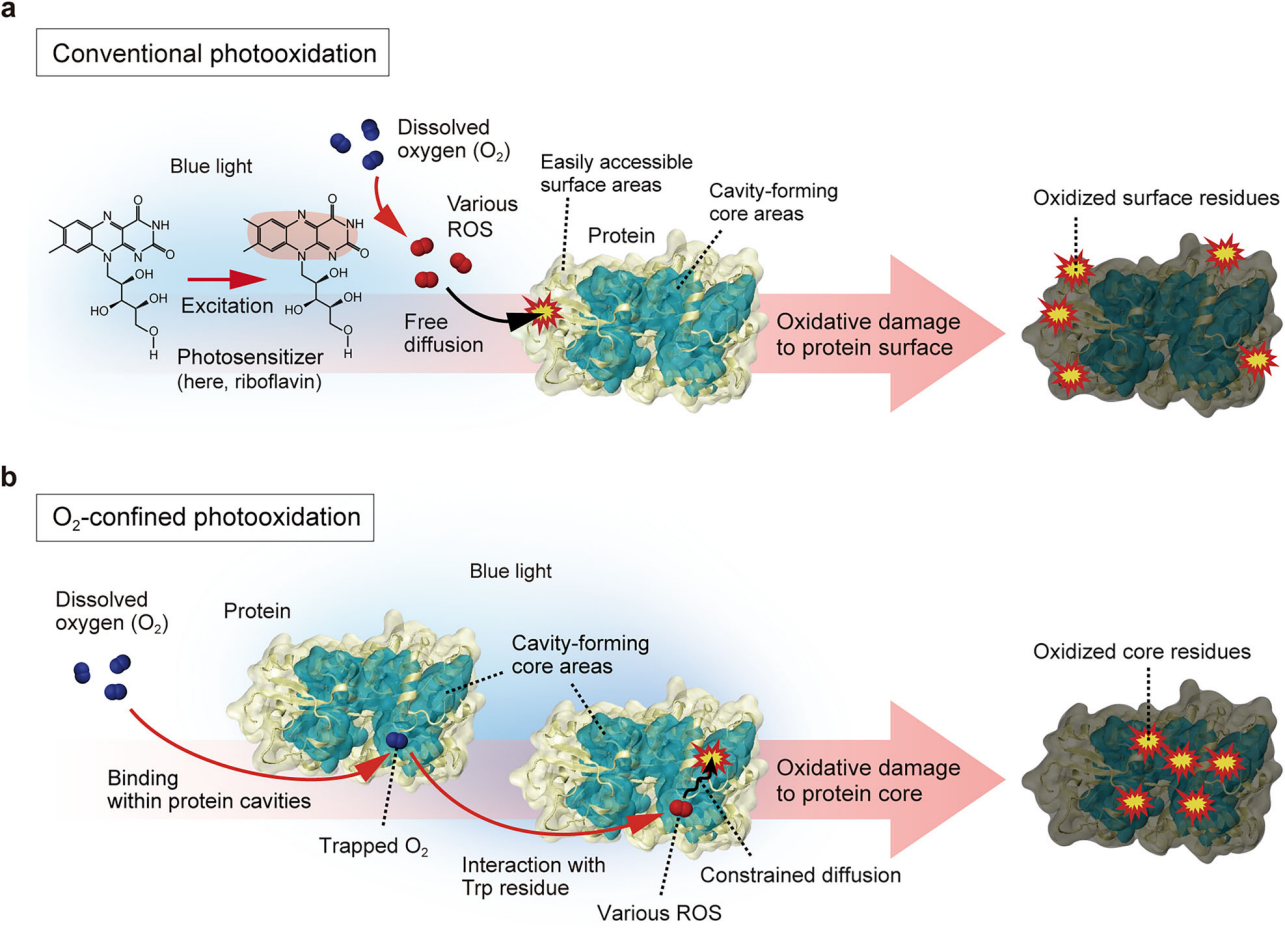
Oxidative protein damage pathways under blue light. **a**, Conventional photooxidation pathway. Blue light excites endogenous PSs (for example, riboflavin), which transfer an electron or energy to dissolved molecular oxygen (O_2_). These photochemical reactions generate ROS, such as superoxide radical (O_2_^•–^), hydroxyl radical (^•^OH) and singlet oxygen (^1^O_2_). These freely diffusible ROS primarily oxidize easily accessible protein surface areas. The isoalloxazine moiety (red) of riboflavin is responsible for light absorption. **b** O_2_-confined photooxidation pathway. Dissolved O_2_ becomes confined within protein cavities that contain Trp residues, acting as endogenous PSs. Upon blue light exposure, Trp mediates the generation of ROS—mainly ^1^O_2_ and ^•^OH—through interactions with the trapped O_2_. The resulting ROS primarily oxidize nearby residues within the protein interior through constrained diffusion. The protein structure shown is maltose-binding protein (MBP), one of the model proteins used in the referenced study^19^. Panel **b** is adapted with permission from ref. ^19^.

**Fig. 5:**
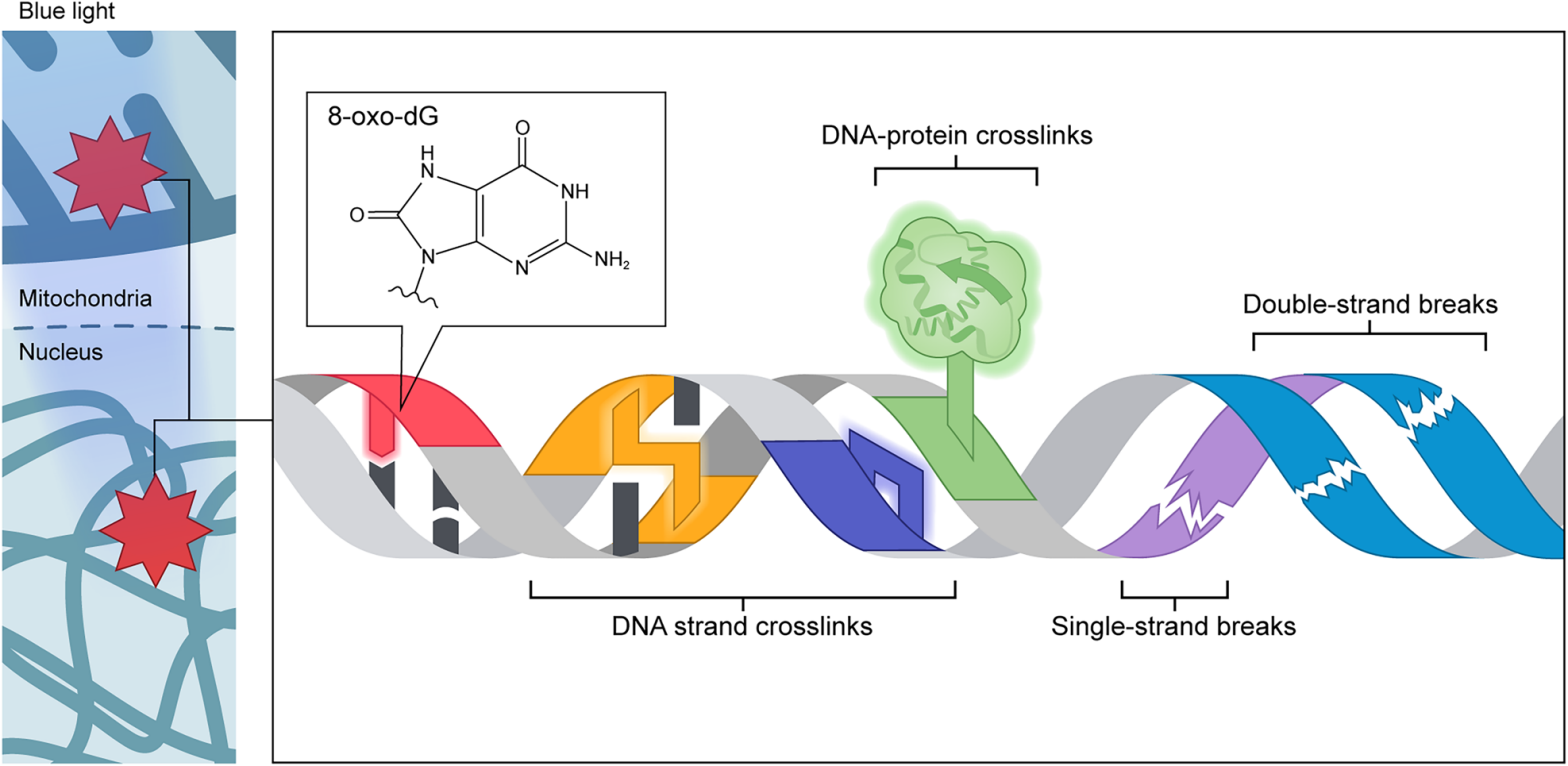
DNA damage under blue light exposure. A schematic diagram illustrating various forms of DNA damage in the nucleus and mitochondria reported under blue light exposure. These include oxidative base modifications (for example, 8-oxo-dG), DNA strand crosslinks, DNA-protein crosslinks, and single- or double-strand breaks.

**Fig. 6:**
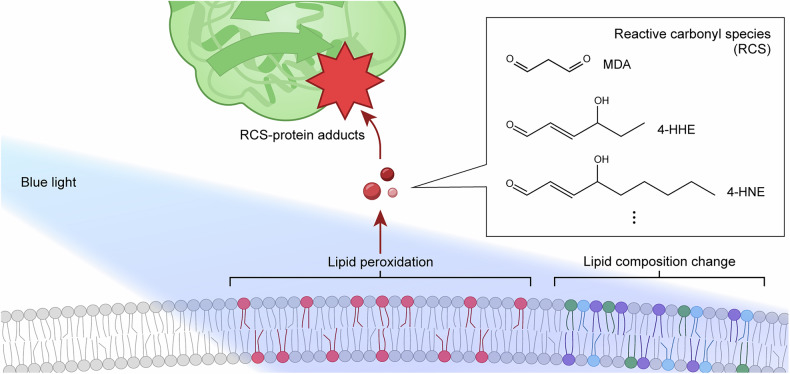
Lipid damage under blue light exposure. A schematic diagram illustrating various types of lipid damage reported under blue light exposure. These include lipid peroxidation, reactive carbonyl species (RCS) generation and lipid composition change (lipid remodeling). RCS can form covalent adducts with cellular proteins, known as RCS–protein adducts.

Photooxidative stress induced by blue light leads to a range of chemical modifications in key biomolecules (Figs. 4–6 and Table 1), primarily mediated by endogenous PSs described in the previous section. These molecular alterations can contribute to diverse cellular and tissue-level effects (Fig. 1). Under blue light exposure, DNA can undergo strand breaks and oxidative base modifications such as 8-oxo-2′-deoxyguanosine (8-oxo-dG), which have been associated with cell cycle arrest^136–138^, activation of autophagy^137,139,140^ and apoptosis-mediated cell death^140–144^. Lipid peroxidation can compromise membrane integrity^120,137,145^ and promote the accumulation of lipid-derived reactive species^146–148^, processes closely linked to blue light-induced ferroptosis^108,149–151^. Proteins are also susceptible to blue light-induced oxidative modifications, which can impair their structure and function, contributing to disrupted proteostasis and an increased burden on protein quality control systems^136,140,152^. These molecular-level changes collectively disrupt overall cellular homeostasis under blue light exposure. This section focuses specifically on the chemical and structural modifications of major biomolecules caused by photooxidative stress under blue light (Figs. 4–6 and Table 1).

### Protein oxidative damage under blue light

The structural diversity of amino acid side chains in proteins leads to residue-specific oxidation patterns, with each residue undergoing distinct chemical modifications based on its intrinsic reactivity, spatial environment, and the nature of ROS involved^153^. Oxidation reactivity assessments consistently identify the following amino acids as particularly susceptible to oxidation: Trp, methionine (Met), histidine (His), cysteine (Cys), tyrosine (Tyr), lysine (Lys) and arginine (Arg) (refs.^2,154^). Their susceptibility is attributed to shared structural features—such as aromatic (for example, Trp and Tyr), sulfur-containing (for example, Met and Cys) or basic nitrogen-containing (for example, His, Lys and Arg) side chains—that are preferentially targeted by specific ROS. Readers are referred to previous review articles for general information on the oxidative modifications of free amino acids and amino acid residues in proteins^29,32,155^.

Early investigations into light-induced oxidation were conducted using broad-spectrum light sources in the presence of PSs, such as proflavine^156,157^. In one study employing chromatographic separation of photodamage products and spectrophotometric detection of absorbance changes, free Trp and Met—as well as their residues within short peptides (di- or pentapeptides)—underwent selective and substantial oxidation upon exposure to incandescent light (typically spanning the visible to infrared range; 150 W, up to 30 h) in acidic proflavine-containing solutions^156^. Notably, free Trp was found to form both melanin and kynurenine (Kyn) as photooxidation products, whereas Trp residues in peptides were predominantly converted to Kyn, with yields reaching up to ~98%. Expanding this work to the protein level, the same group examined photooxidation in lysozyme irradiated at wavelengths above 320 nm in the presence of proflavine and formic acid, using a segmented-flow autoanalyzer and a spectrophotometer^157^. Irradiation led to a marked decrease in absorbance at 280 nm—indicative of Trp loss—and the emergence of new peaks near 258 nm and 360 nm, consistent with the spectral signature of Kyn reported in their earlier study^156^.

Subsequent studies explored protein photooxidation under well-defined blue light irradiation conditions^158,159^ (Table 1). One such investigation systematically examined how varying parameters influence protein oxidation: light wavelength (345, 365, 449, 556 and 621 nm), PSs (for example, riboflavin, rose bengal and methylene blue) and solvent environments (for example, phosphate buffer at pH 7.0 and 50% acetic acid)^158^. Among the tested conditions, lysozyme samples containing riboflavin at a 2:1 molar ratio exhibited selective oxidation of Trp and His residues when irradiated with blue light at 449 nm (9 × 10^5 ^J/mol). To evaluate residue-specific oxidative changes, irradiated samples were subjected to acid hydrolysis and compared with nonirradiated controls. The resulting amino acid mixtures were analyzed using a segmented-flow autoanalyzer, enabling quantification of individual residues. No detectable oxidation occurred in the absence of riboflavin, underscoring its essential role as a PS in light-induced protein oxidation^158^.

Further investigations examined blue light-induced photooxidative damage using the structurally homologous proteins, α-lactalbumin and lysozyme, both irradiated at 452 nm (2 W/m^2^, up to 4 h) in the presence of riboflavin^159^. This study focused on evaluating the influence of structural context by comparing proteins under native and denatured conditions, with the latter induced by urea treatment. Residue-specific photooxidative reactivity was assessed by monitoring time-dependent loss of amino acids following acid hydrolysis. From these measurements, the kinetic results of amino acid loss expressed as quantum yields were derived to compare the extent of photooxidation across different structural states. Among the residues analyzed, Trp exhibited the most pronounced photooxidative loss regardless of structural conditions, followed by Tyr and His. The variation in residue-specific oxidation, along with protein structural states, suggests that blue light-induced photooxidative damage is governed not only by the intrinsic redox susceptibility of residues but also by their structural context, such as spatial positioning and solvent accessibility.

On the basis of previous studies, freely diffusing ROS are thought to preferentially target redox-sensitive residues exposed on protein surfaces^160–163^ (Fig. 4a). While this has led to the common assumption that oxidation is largely confined to surface-exposed sites, proteins also contain internal solvent-accessible cavities that allow entry of small molecules, including O_2_ and ROS. A recently identified pathway—termed O_2_-confined photooxidation—has revealed that protein damage can also originate from these cavity-containing interiors^19^ (Fig. 4b). In this pathway, O_2_ is initially trapped within protein cavity sites, where it transiently forms a light-absorbing complex with nearby Trp residues. Upon blue light exposure at 447 nm (9.2 or 16 mW/cm^2^, up to 12 h), this molecular complex generates ROS such as ^1^O_2_ and ^•^OH within the protein interior. Owing to spatial confinement, these ROS engage in localized diffusion and oxidatively modify buried residues. This work uncovers a previously unrecognized route of blue light-induced protein damage, highlighting the role of internal protein architecture in modulating oxidative susceptibility.

Cell-based studies conducted under short-wavelength visible light (390–550 nm) have shown that protein damage—characterized by protein carbonylation—arises not only from direct ROS-mediated oxidation but also through secondary oxidative modifications involving reactive carbonyl species such as methylglyoxal and lipid peroxidation byproducts^145,164–167^. To quantify these carbonyl modifications, various antibody-based immunodetection techniques targeting modified sites have been employed. In one study, cultured human RPE cells supplemented with lipofuscin granules were irradiated with visible light (390–550 nm, 2.8 mW/cm^2^) for 24 or 48 h, resulting in a marked increase in protein carbonylation, particularly within membrane protein fractions^164^. Similarly, significant protein carbonyl accumulation was observed in ARPE-19 cells (derived from human RPE cells) following blue light exposure (430 nm, 1.5 mW/cm^2^, 20 min) in the presence of accumulated A2E, with protein carbonyl levels increasing by up to several dozen times relative to nonirradiated controls^165^. Reactive carbonyl species such as methylglyoxal can specifically modify amino acid residues such as arginine, as evidenced by elevated levels of methylglyoxal-derived adducts such as methylglyoxal-H1. These adducts can subsequently undergo further modifications, including protein crosslinking^168^. Readers are referred to the ‘Lipid oxidative damage under blue light’ section for details on protein modifications caused by lipid peroxidation byproducts.

Recent advances in mass spectrometry-based proteomics have enabled more detailed and direct identification of blue light-induced oxidative modifications in cells^46,152^. In one study, a chemoproteomic approach using Cys-specific labeling was applied to *Drosophila* eye tissue—a model system with rhodopsin-based photosensing—following blue light exposure (465 nm, 2 mW/cm^2^, 8 h)^152^. Oxidative modifications were detected at numerous Cys residues, including those located within catalytic sites, ligand-binding motifs, and structural domains critical for maintaining protein conformation. These findings suggest that such modifications may disrupt enzymatic activity and compromise structural integrity. Notably, the study also revealed that Cys oxidation patterns and the set of oxidized proteins varied across different oxidative stress conditions (for example, blue light exposure, aging and H_2_O_2_ treatment), underscoring the context-dependent nature of the redox proteome. In another chemoproteomic study, blue light-induced oxidative modifications were identified at Tyr and His residues in multiple mammalian cell types, including human cervical cancer cells (HeLa), murine melanoma cells (B16F10) and human corneal epithelial cells (iHCEC, a physiologically relevant light-exposed model)^46^. In cells supplemented with flavin derivatives, blue light exposure (450 nm, 14 mW/cm^2^, 10 min) led to oxidative damage that was predominantly observed in membrane-associated proteins, particularly integrins, compared to intracellular proteins. Specifically, oxidation of integrin β1 impaired cell adhesion, as indicated by a blue light-induced loss of its active conformation detected via immunostaining, a change not observed under H_2_O_2_ treatment.

### DNA oxidative damage under blue light

Although early research predominantly focused on UV-induced DNA damage, studies beginning in the 1970s revealed that blue light can also induce nonenzymatic DNA strand breaks and other oxidative lesions (Fig. 5 and Table 1). Initial investigations often involved supplementation with endogenous PSs^79,169^. For example, one study demonstrated that blue light (415–465 nm, 1.7 mW/cm^2^, 2 h) induced a range of photochemical alterations in DNA in the presence of riboflavin^169^. Among nucleosides, deoxyguanosine exhibited marked changes in UV absorbance following blue light exposure. In isolated calf thymus DNA, additional effects were observed, including altered absorbance spectra, reduced thermal stability, and changes in sedimentation and buoyant density. DNA fragmentation via strand breaks was also detected in HeLa cells, as indicated by reduced migration in alkaline sucrose gradient centrifugation. Similarly, blue light exposure (420–490 nm, 16.7 W/m^2^, up to 50 kJ/m^2^, up to 50 min) caused DNA strand breaks in normal human fibroblasts (derived from skin biopsies) supplemented with bilirubin^79^. This study further showed that irradiation of bilirubin generated H_2_O_2_, which probably contributed to DNA damage, as similar strand breaks were detected in cells incubated in the dark with pre-irradiated bilirubin.

Blue light-induced DNA strand breaks have also been observed in the absence of externally supplemented PSs^170,171^. In one study, human epithelial carcinoma cells (KB cells) were exposed to fluorescent light either in the visible-near-infrared range (400–700 nm, 141 μW/cm^2^, 15.2 kJ/m^2^) or the blue range (450 nm, 652 μW/cm^2^, up to 70.4 kJ/m^2^), without any added PSs^170^. Treated cells exhibited slower DNA migration in alkaline sucrose gradient centrifugation, indicating the presence of strand breaks. Further studies using various mammalian cell lines—including human embryonal carcinoma (P3), human skin keratinocytes (HaCaT), mouse leukemia cells (L1210) and Chinese hamster ovary (CHO)-derived reporter cells (AS52)—confirmed that blue light (400–500 nm, up to 42 kJ/m^2^) induces multiple types of DNA damage, including strand breaks^171^. Alkaline elution assays combined with DNA repair endonucleases revealed that while blue light can cause single-strand breaks (SSBs), it more prominently induces oxidative base modifications, such as (8-oxo-dG).

8-Oxo-dG has emerged as one of the principal oxidative DNA lesions induced by blue light exposure. Early studies identified deoxyguanosine as particularly susceptible to photooxidation under blue light owing to guanine’s uniquely low one-electron reduction potential—the lowest among the canonical DNA bases—which renders it highly prone to oxidative modifications^169^. Once formed, 8-oxo-dG can mispair with adenine during replication, resulting in G:C to T:A transversion mutations that compromise genomic integrity^172^. Initial investigations using broad-spectrum light sources that included the blue region demonstrated 8-oxo-dG formation. In one such study, visible to near-infrared irradiation (100 W tungsten lamp, up to 4 min) of isolated calf thymus DNA in the presence of riboflavin led to the generation of 8-oxo-dG (ref. ^173^). Isotope labeling experiments implicated an oxidative mechanism involving electron transfer from guanine to excited-state riboflavin, forming a guanine radical cation that finally undergoes oxidation at C8. Extending these findings to cellular models, another study showed that visible to near-IR light (150 W tungsten lamp, up to 30 min) induced 8-oxo-dG formation in mouse lymphoma cells and human lung fibroblasts supplemented with riboflavin^174^. Quantification by high-performance liquid chromatography coupled with electrochemical detection (HPLC–ECD) revealed a proportional increase in 8-oxo-dG levels with increasing riboflavin concentrations and irradiation durations.

More targeted wavelength studies have further substantiated the formation of 8-oxo-dG under blue light exposure. In one study, CHO-derived reporter cells (AS52) were irradiated with filtered monochromatic light (within the 290–600 nm range, 1–4 W/m^2^, up to 150 kJ/m^2^), revealing that DNA excision by the repair enzyme Fpg—primarily recognizing 8-oxo-dG—peaked in the blue region between 400 and 450 nm, with a maximum at 420 nm (ref. ^175^). In another investigation, 8-oxo-dG formation was assessed across filtered monochromatic wavelengths (within the 313–434 nm range, up to 1,000 kJ/m^2^) in various human skin cell types, including primary fibroblasts (FEK4), lymphoblastoid cells (TK6) and melanoma cells (GLL19)^176^. HPLC–ECD analysis in FEK4 cells showed the highest yield of 8-oxo-dG at 365 nm, with substantial oxidation also observed at 405 nm and 434 nm. Furthermore, under broad UVA/blue light irradiation (350–450 nm, 600 kJ/m^2^) in the presence of D_2_O—which prolongs the lifetime of ^1^O_2_—8-oxo-dG levels increased by approximately fourfold, supporting the involvement of ^1^O_2_ in its formation alongside direct photooxidation via light absorption. A recent study using an alkaline comet assay combined with Fpg also revealed substantial Fpg-mediated DNA excision in human skin keratinocytes (HaCaT) following blue–violet light exposure (408 nm, 50 J/cm^2^), with a lesser but still evident level of excision observed under blue–turquoise light (466 nm, 100 J/cm^2^)^177^.

While cyclobutene pyrimidine dimers (CPDs)—covalent photoproducts formed between adjacent pyrimidine bases—are well established as canonical DNA lesions resulting from UV irradiation, their association with blue light remains controversial^142,176,178–181^. In one study, researchers compared DNA lesions induced by blue light (415 nm, 8 mW/cm^2^, up to 14.4 J/cm^2^) with those caused by UV or UV–visible light (UVA–visible: 290–700 nm, 76.5 mW/cm^2^, 15 J/cm^2^; UVB: 312 nm, 0.91 mW/cm^2^, 0.08 J/cm^2^) in human epidermal keratinocytes (ScienCell)^178^. Using comet assay combined with T4 endonuclease V (T4 Endo V)—which converts CPDs into SSBs, thereby increasing comet tail moments under alkaline conditions—they observed a light dose-dependent increase in DNA fragmentation following blue light exposure. The extent of damage closely paralleled that observed with UVB-induced CPD formation. In a separate study, blue light was also shown to compromise CPD repair^179^. Specifically, pre-exposure to blue light (427 ± 30 nm, 257.4 W/m^2^, 60 J for 39 min or 92 J for 1 h) before UVB irradiation significantly reduced the efficiency of CPD removal in a reconstructed human epidermis model, as assessed by HPLC–tandem mass spectrometry (HPLC–MS/MS). By contrast, UVB exposure alone led to progressive CPD repair over a 48-h period.

However, several studies have reported conflicting results regarding CPD formation under blue light exposure^142,180,181^. In one investigation using murine melanoma cells (B16F1), CPDs were not detected following blue light irradiation (465 nm, 10 or 50 W/m^2^, up to 24 h), as assessed by anti-CPD immunofluorescence, whereas substantial CPD formation was observed under UV light exposure^142^. Similarly, a study examining the UV–visible boundary region (385–405 nm) in human skin keratinocytes (HaCaT) found that CPD formation—evaluated via immunohistochemistry–immunofluorescence and HPLC–MS/MS—occurred at 385 nm in a light dose-dependent manner but was minimal or absent at 405 nm^180^. In addition, in human skin irradiated at 385 nm (74 mW/cm^2^, up to 150 J/cm^2^), CPD levels increased over a 2-h period and remained elevated for up to 24 h. By contrast, 405 nm exposure (260 mW/cm^2^, up to 150 J/cm^2^) resulted in only limited CPD detection without a consistent temporal pattern, suggesting a substantially lower CPD-inducing potential. More recently, another study reported that blue light exposure (417 ± 5 nm, 6.9 mW/cm^2^, up to 60 J/cm^2^) did not induce detectable levels of CPDs in human dermal fibroblasts^181^. ELISA and immunoblotting analyses consistently confirmed the absence of this DNA lesion across all tested conditions.

Visible light exposure, including wavelengths in the blue region, has also been linked to the formation of DNA–protein crosslinks (DPCs)^182,183^. In one study, mouse embryonic and lung cells exposed to fluorescent light (400–700 nm, 4.6 W/m^2^, up to 20 h) exhibited delayed DNA elution in alkaline elution assays compared to unexposed controls, suggesting the presence of DNA crosslinks and/or DPCs^182^. A follow-up study by the same group confirmed that DPCs were the dominant crosslinking species under fluorescent light (400–700 nm, with or without wavelength filters; 4.6 or 8.6 W/m^2^, up to 20 h), as evidenced by their sensitivity to proteinase K treatment in the elution assay^183^. This effect was consistently observed across various mice and human cell types. The researchers further identified 450–520 nm as the most effective wavelength range for inducing DPCs, with crosslink formation enhanced under high-oxygen conditions.

The mechanism of DPC formation may vary depending on the wavelengths of light exposure. In one study, P3 cells were irradiated with monochromatic light across a broad spectral range (254–545 nm, up to 710 W/m^2^, up to 4,018 kJ/m^2^), and DPC formation was quantified using the alkaline elution assay with proteinase K treatment^184^. Apparent DPC levels were measured as a function of fluence at each wavelength, leading to an action spectrum for DPC induction. Two prominent peaks emerged in this spectrum: one in the UVC region (254 nm) and another in the blue region (405 nm), suggesting that distinct mechanisms may be involved at each wavelength. In a follow-up study, the same group showed that DPC formation at 405 nm was markedly reduced under oxygen-depleted conditions but increased in D_2_O, which prolongs the lifetime of ^1^O_2_ (ref. ^185^). By contrast, these treatments had no effect on DPC induction at 254 nm. Together, these findings suggest that ROS, particularly ^1^O_2_, play a greater role in DPC formation under blue light irradiation.

Visible light, particularly within the blue spectrum, has been shown to induce higher-order chromatin and chromatid damage, including chromatin condensation, chromatid breaks, sister chromatid exchanges (SCEs), and micronucleation^138,142,171,178,182,186–188^. In murine melanoma cells (B16F1), chromatin condensation was observed following blue light exposure (465 nm, 10 or 50 W/m^2^, up to 24 h), visualized using Hoechst 33342 staining^142^. One study demonstrated that fluorescent light (400–700 nm, 4.6 W/m^2^, up to 20 h) induced various chromosomal damage in mouse embryonic and lung cells^182^. Karyotype analysis revealed increased frequencies of chromatid breaks, minute fragments, and chromatid exchanges, ranging from approximately 3- to 26-fold higher than in controls. Further research showed that blue light (420–500 nm, 3.61 W/m^2^, up to 300 kJ/m^2^) was particularly effective at inducing SCEs in V-79 fibroblasts (derived from CHO cell line) in a light dose-dependent manner^187^. Similar increases in SCE frequency were observed in CHO cells following blue light exposure (425–475 nm, 1–5 μW/cm^2^/nm, up to 24 h), with increases of twofold or more^138^. Notably, neonates with Down syndrome exhibited higher SCE frequencies under blue and green light exposure (480 and 510 nm, two 20 W lamps, up to 6 h) compared to healthy controls^188^. In human epidermal keratinocytes (ScienCell), blue light exposure (415 nm, 8 mW/cm^2^, up to 14.4 J/cm²) led to a dose-dependent increase in micronucleated cells, which are extranuclear structures containing damaged chromosome fragments or missegregated whole chromosomes^178^.

Subcellular localization can influence susceptibility to blue light-induced DNA damage^50,177^. Among subcellular organelles, mitochondria are particularly vulnerable owing to their high levels of blue light-sensitive endogenous PSs, such as flavin, and the absence of protective histones^189^. In one study using human RPE cells, the susceptibility of mitochondrial DNA (mtDNA) and nuclear DNA (nDNA) to blue light-induced damage (390–550 nm, 2.8 mW/cm^2^, up to 6 h) was evaluated by qPCR^50^. Following blue light irradiation, a marked decrease in mtDNA amplification efficiency was observed (peaking at 3 h, ~0.7 lesions/10 kb mtDNA), whereas no changes were detected in nDNA. Although the specific DNA lesions were not identified, the heightened vulnerability of mtDNA was further supported by experiments using isolated mitochondria, where blue light exposure led to a time-dependent increase in ROS production, such as O_2_^•–^, ^1^O_2_ and ^•^OH. A more recent study using human skin keratinocytes (HaCaT) conducted a wavelength-specific analysis of mtDNA across the visible light spectrum^177^. Compared to longer wavelengths, blue–violet light (408 nm, 50 J/cm^2^) and blue–turquoise light (466 nm, 100 J/cm^2^) caused the most pronounced reductions in mtDNA amplification efficiency by ~40% and ~25%, respectively.

Recently, DNA sequencing-based techniques have enabled genome-wide, high-resolution mapping of DNA damage, including DNA strand breaks and oxidative base modification^190,191^. Methods such as BLESS, BLISS and DSBCapture are designed to detect double-strand breaks (DSBs), while SSiNGLe, GLOE-seq and SAR-seq are specific for SSBs. For oxidative base modifications such as 8-oxo-dG, approaches including Oxidip-seq, OG-seq and Click-code-seq have been developed, often utilizing damage-specific antibodies, biotin-streptavidin pulldown or click-chemistry-based probes^190,191^. These advanced sequencing strategies have greatly expanded our capacity to map the genomic distribution of DNA lesions, identify preferential sites of damage accumulation and evaluate their impact on gene expression^192,193^. These technologies could be further leveraged to obtain comprehensive genome-wide profiles of DNA damage induced or enhanced by blue light irradiation.

### Lipid oxidative damage under blue light

Compared to saturated and monounsaturated fatty acids, ROS preferentially target polyunsaturated fatty acids (PUFAs), which comprise a substantial fraction of membrane phospholipids in eye tissues^194^. PUFAs are particularly susceptible to hydrogen abstraction and radical chain reactions, making them primary substrates for lipid peroxidation^133^. Specifically, under oxidative stress, hydrogen abstraction from a methylene group between double bonds generates a lipid radical that rearranges into a conjugated diene—detectable by UV spectrophotometry^195^. The diene then reacts with O_2_ to form a lipid peroxyl radical, which can abstract a hydrogen atom from a neighboring PUFA, producing a lipid hydroperoxide—detectable by the FOX assay, chemiluminescence or iodometric assay^195^. The lipid peroxyl radicals or hydroperoxides can further degrade into reactive carbonyl species, such as malondialdehyde (MDA), 4-hydroxynonenal (4-HNE) and 4-hydroxyhexenal (4-HHE), which are commonly detected by the TBARS assay or immunochemical methods^196–199^. These species can form covalent adducts with DNA, thereby compromising genomic integrity^200^. For example, MDA predominantly forms exocyclic DNA adducts at deoxyguanosine residues, yielding MDA-deoxyguanosine adduct (M1dG)^201^. Lipid peroxidation products can also form covalent adducts with proteins, primarily at cysteine, histidine and lysine residues^202^. Readers are referred to previous review articles for general information on lipid peroxidation and relevant analytical assays^195,203^.

The mechanistic understanding of lipid peroxidation provides a framework for studying light-induced lipid damage (Fig. 6 and Table 1). In one study, albino rats were exposed to cool white fluorescent light (400–700 nm, 1 or 5 klux, 3 h) to examine the formation of 4-HNE- or 4-HHE-protein adducts using dot blot assay and immunohistochemistry^197^. Following 5 klux light exposure, both adduct types increased significantly in the retina and RPE (1.7- and 1.5-fold for 4-HNE; 1.7- and 1.8-fold for 4-HHE), while only 4-HHE adduct levels increased in rod outer segments (1.4-fold). These findings suggest that intense visible light enhances lipid peroxidation, leading to secondary protein modifications in retinal tissues. In a follow-up study by the same group, intense white light exposure (400–700 nm, 5 klux, 3 h) in albino rats resulted in elevated levels of 4-HNE-protein adducts in the retina^204^. Proteomic analysis using 2D gel electrophoresis, western blotting and peptide mass fingerprinting revealed that the modified proteins were involved in key metabolic pathways, including energy metabolism and phototransduction.

Similarly, light irradiation at a specific wavelength within the blue spectrum also promoted lipid peroxidation in retinal tissues, as indicated by increased levels of 4-HNE-protein adducts^166^. In this study, bovine RPE cells were irradiated with blue LED light at 470 nm (4.8 mW/cm^2^,1–50 J/cm^2^) and lipid peroxidation was assessed using a 4-HNE-specific immunochemical assay. In addition, intracellular ROS levels were also measured using the DHR-123 assay, which detects a broad range of ROS, including O_2_^•–^, H_2_O_2_ and ^•^OH (ref. ^205^). Under 50 J/cm^2^ exposure, 4-HNE-protein adduct levels increased by up to approximately sevenfold compared to dark controls, accompanied by a marked rise in intracellular ROS. Therefore, blue light appears to enhance ROS generation, probably facilitating 4-HNE modification of cellular proteins.

Endogenous pigments such as lipofuscin and melanosomes can contribute to blue light-induced ROS generation and lipid peroxidation in retinal tissues^88,206^. In one study, lipid peroxidation within isolated lipofuscin from human RPE cells was assessed using iodometric and TBARS assays^88^. Following blue light exposure (408–495 nm, 220 mW/cm^2^, up to 70 min), levels of lipid hydroperoxides in lipofuscin increased sharply, followed by a gradual decline. By contrast, MDA showed a continuous, gradual increase, probably reflecting the degradation of lipid hydroperoxides during their decline phase. Lipid peroxidation may occur not only within large pigment granules but also in nearby PUFAs, probably through pigment-mediated photooxidative reactions. The photooxidative potential of various RPE pigments—lipofuscin, melanosomes and melanolipofuscin—was assessed using the NADPH-glutathione peroxidation assay or TBARS assay with substrate PUFAs such as docosahexaenoic acid (22:6; DHA, an omega-3 fatty acid abundant in retina) and linoleic acid (18:2) (ref. ^206^). In this study, pigment–PUFA mixtures were irradiated with a blue–green argon laser (488 and 514 nm, 50–1,500 mW/cm^2^, 10 min). Under this light condition, DHA exhibited a sharp increase in lipid hydroperoxide levels up to a light intensity of 400 mW/cm^2^. Lipid peroxidation levels were highest in the presence of melanosomes, followed by lipofuscin and melanolipofuscin. By contrast, for linoleic acid, overall peroxidation levels were lower and showed a different pigment-related order of effect: lipofuscin, melanosomes and melanolipofuscin.

A recent study further investigated DHA peroxidation in the presence of A2E, a photosensitizing component of lipofuscin^145^. In this work, bovine retina extracts and DHA-supplemented ARPE-19 cells (derived from human RPE cells) were exposed to various light sources—UVA light (350–400 nm, 1 mW/cm^2^), blue light (430 nm, 3 mW/cm^2^) and cool or warm white light (400–700 nm, 5 or 7 mW/cm^2^, respectively)—in the presence or absence of A2E. The resulting DHA-derived peroxidation adducts, such as 4-hydroxy-7-oxohept-5-enoic acid-lactone-glutathione (HOHA-lactone-GSH), 4,7-dihydroxyhept-5-enoic acid-lactone-glutathione (DHHA-lactone-GSH) and carboxyethylpyrrole (CEP), were quantified using LC–MS/MS or immunohistochemistry. In bovine retina extracts, 1 h of visible light exposure (400–700 nm) led to approximately twofold (warm white) and threefold (cool white) increases in HOHA-lactone-GSH levels compared to dark controls. In addition, in DHA-supplemented ARPE-19 cells, DHHA-lactone-GSH levels increased in a time-dependent manner under both blue and UVA light exposure, even in the absence of A2E. In the presence of A2E, blue light exposure further accelerated DHHA-lactone-GSH formation, resulting in an approximately sevenfold increase after 1 h relative to controls without A2E.

Irradiation with visible light—or more specifically, green or blue light—not only induces lipid peroxidation^207^, but also alters the detailed composition of lipid membranes^208–211^. In one of the earliest studies, albino rats were exposed to fluorescent light (400–700 nm, 1,075–1,345 lux, up to 72 h)^208^. Following the light exposure for 72 h, changes in lipid composition were observed in the retina: palmitic acid (16:0) and oleic acid (18:1) increased by 8.6% and 33%, respectively, while DHA (22:6) decreased by 26%. Conjugated dienes, a hallmark of lipid peroxidation, also nearly doubled relative to dark controls. In another study, albino rats were exposed to either continuous or intermittent green light irradiation (490–580 nm, 1,750–2,000 lux, up to 72 h)^209^. After 16 h of exposure, levels of palmitic acid (16:0), oleic acid (18:1), arachidonic acid (20:4) and cholesterol increased by 6%, 4%, 1% and 18%, respectively, while DHA (22:6) content declined by 12%. These findings suggest that exposure to broad-spectrum visible light or green light can trigger reorganization of membrane lipid profiles in retinal tissues.

Recent lipidomic studies have shown that specific blue light irradiation can selectively alter the composition of certain lipid species, despite minimal changes in overall lipid profiles^210^. In one study using COS-7 fibroblast cells (derived from African green monkey kidney fibroblasts), the cells were exposed to blue LED light (465 nm, 1 mW/cm^2^, up to 1 h)^210^, and lipid profiles were analyzed using LC–MS/MS. Following 1 h of blue light exposure, approximately 7% of detected lipid species increased in abundance, while about 3% decreased. Notably, inflammation-associated lipids—including lysophospholipids such as lysophosphatidylcholine (LPC; 16:0, 18:0, 18:1), lysophosphatidylethanolamine (LPE; 18:1), and lysophosphatidylserine (LPS; 16:0, 18:1), and arachidonic acid (20:4)—were upregulated by more than 40%, suggesting potential activation of pro-inflammatory lipid signaling. Conversely, lipids associated with anti-inflammatory effects, such as DHA and eicosatetraenoic acid, also increased by ~42% and ~53%, respectively, suggesting a compensatory or adaptive lipidomic remodeling response.

## Perspectives

With the growing reliance on artificial lighting in modern life, human exposure to blue light has become more frequent and prolonged. Accordingly, research into its physiological effects has expanded across diverse disciplines, including ophthalmology and dermatology^8,212^. However, our understanding across multiple levels—from molecular mechanisms to associated health outcomes—remains incomplete. While numerous studies have reported potential health risks linked to blue light, many experimental setups involve exposure intensities that far exceed those encountered in everyday environments^1,213^. Moreover, the lack of longitudinal studies limits our understanding of its long-term effects on human health^1,214^. At the molecular level, further investigation is needed to clarify the mechanisms underlying blue light-induced damage. For example, newly proposed pathways such as O_2_-confined photooxidation add complexity to the landscape of photooxidative damage in blue light-exposed cells and tissues^19^. Recent studies on photoinduced molecular damage also challenge earlier reports, suggesting that CPDs may not be a major form of DNA damage under blue light exposure^142,180,181^. Advances in analytical technologies continue to expand the boundaries of our understanding of photooxidative molecular damage. For instance, omics approaches—such as redox-sensitive proteomics, genome-wide next-generation DNA sequencing and lipidomic profiling—are increasingly used to characterize oxidative modifications or compositional changes in greater detail^46,152,190,191,210^. These methods offer new opportunities to refine mechanistic links between molecular damage, cellular and tissue dysfunction, and long-term health outcomes associated with blue light exposure.
